# Discrimination of Deciduous Tree Species from Time Series of Unmanned Aerial System Imagery

**DOI:** 10.1371/journal.pone.0141006

**Published:** 2015-11-24

**Authors:** Jonathan Lisein, Adrien Michez, Hugues Claessens, Philippe Lejeune

**Affiliations:** 1 Laboratory of Forest Resources Management, Department of Biosytem Engineering, University of Liège—Gembloux Agro-Bio Tech. 2, Passage des déportés, 5030 Gembloux, Belgium; 2 Ecole nationale des sciences géographiques, 6 et 8 avenue Blaise Pascal, Cité Descartes, Champs-sur-Marne, 77455 Marne la Vallée, France; University of Verona, ITALY

## Abstract

Technology advances can revolutionize Precision Forestry by providing accurate and fine forest information at tree level. This paper addresses the question of *how* and particularly *when* Unmanned Aerial System (UAS) should be used in order to efficiently discriminate deciduous tree species. The goal of this research is to determine when is the best time window to achieve an optimal species discrimination. A time series of high resolution UAS imagery was collected to cover the growing season from leaf flush to leaf fall. Full benefit was taken of the temporal resolution of UAS acquisition, one of the most promising features of small drones. The disparity in forest tree phenology is at the maximum during early spring and late autumn. But the phenology state that optimized the classification result is the one that minimizes the spectral variation within tree species groups and, at the same time, maximizes the phenologic differences between species. Sunlit tree crowns (5 deciduous species groups) were classified using a Random Forest approach for monotemporal, two-date and three-date combinations. The end of leaf flushing was the most efficient single-date time window. Multitemporal datasets definitely improve the overall classification accuracy. But single-date high resolution orthophotomosaics, acquired on optimal time-windows, result in a very good classification accuracy (overall out of bag error of 16%).

## Introduction

### Context

In the field of environmental sciences, remote sensing techniques are currently undergoing a revolution [1]. Although remote sensing data has been used for a long time to study ecological phenomena, traditional spaceborne and airborne imagery have failed to provide convenient information at a fine temporal and spatial scale [1]. Thanks to rapid technological advances, a large upsurge in the development of *civil* unmanned aerial systems (UAS) has changed the story. Unmanned aerial systems, also called unmanned aerial vehicles or drones, are new platforms that come in various configurations. Small drones devoted to mapping purposes are versatile, cost effective and flexible. They operate on users’ demand and deliver very high resolution images when used with an onboard optical sensor. Environmentalists have now the opportunity, at a reasonable price, to follow the development of ecological phenomena on a local scale by means of multitemporal datasets of outstanding spatial resolution.

Precision Agriculture, devoted to the study of temporal and spatial variations in agricultural production, is expected to largely benefit from UAS technology [2]. Similarly, *precision forestry* can take advantage of mapping drones in order to analyze and monitor forest ecosystems on a tree-level, instead of on a stand-level [3]. In forest inventory information, the forest composition is essential since tree species influence, to a large extent, numerous other forest characteristics (e.g. biomass, biodiversity, tree damage). Management of mixed and uneven-aged forests could therefore particularly benefit from tree-based inventory resulting from multi-source forest inventory (combination of field inventory and remote sensing data). However, mapping tree species with optical imagery remains a difficult task as the spectral variation within species may be greater than between them [4–6].

### Discrimination of forest species by remote sensing

**Fig 1:**
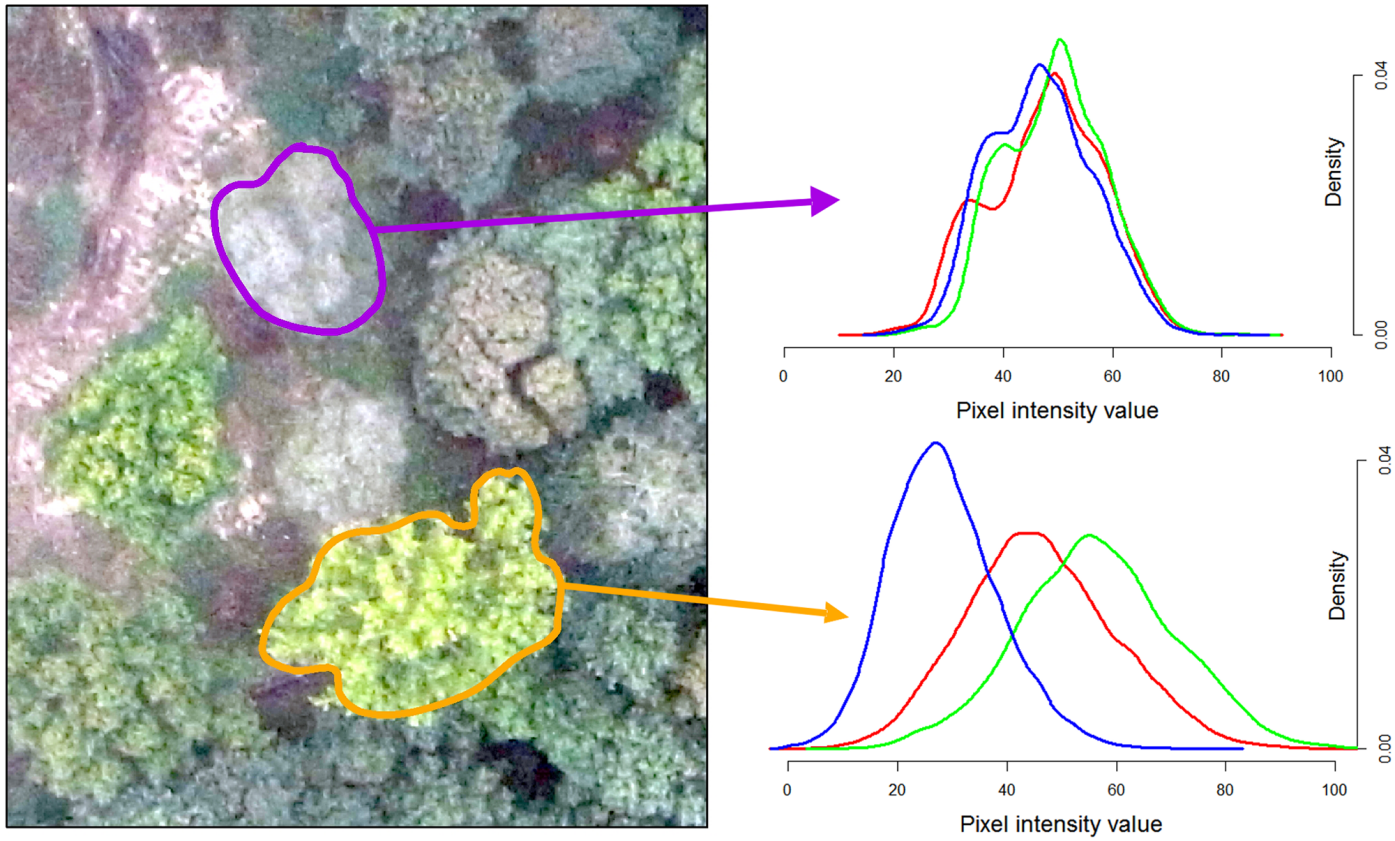
Differences in spectral response captured with a compact camera mounted on an UAS for two tree crowns (birch and poplar species). Density curves for the red, green and blue band are depicted on the right.

The determination of forest composition by remote sensing is based on the principle that every tree species has its own spectral signature. Sensors such as a Red-Green-Blue camera enable the measurement of the spectral response of a tree across a specific wavelength range of the electromagnetic radiation. Discrimination of tree species takes advantage of the differences in spectral response between each tree. UAS imagery of very high spatial resolution—up to centimetric resolution—is very promising for the discrimination of forest species [7]. The Fig 1 illustrates the difference in spectral response between a birch and a poplar on an aerial image acquired by drone.

Remote sensing is proven to be an effective approach for the tracking of phenological changes [8]. Yet, no advantage has been taken of the benefits of UAS flexibility to study *the timing of recurring biological events* occurring in forest ecosystems. The evaluation of seasonal spectral separability among plant species has been previously studied at diverse scales, from forest stand [9, 10] to plant leaf [6, 11, 12]. Multiple platforms and sensors have been used for this purpose, from consumer-grade RGB camera to hyperspectral sensor. Unfortunately, no general agreement was found to determine at best the optimal phenology time-windows for tree species discrimination. Numerous studies on forest composition have been conducted on the basis of multispectral airborne and spaceborne data. However, the temporal variation of species phenology was generally not taken into consideration [13]. For example, Immitzer et al. [14] used a single-date spaceborne image (acquisition date: 10 July, GSD of 50 cm for panchromatic image and of 2 m for multispectral bands) to differentiate the individual sunlit tree crowns of 10 species (5 deciduous and 5 conifers). With an object-based image analysis approach, they ended up with a very promising overall classification accuracy of 82%. In addition, these authors have reviewed past research on the discrimination of temperate forest species, based on monotemporal remote sensing data. The resulting classification scenarios, based on monotemporal acquisitions, are however barely comparable since species discrimination varies with the phenology stage, the species of interest, and the utilized sensor.

On the other hand, Hill et al. [5] used a time series of 5 multispectral Airborne Thematic Mapper images (11 bands of 2m spatial resolution) in order to classify 6 broadleaved species. The class separability of temperate deciduous tree species, at the crown level, was shown to increase when using multitemporal data. The optimal three-date combinations for 6 broadleaved species led to an overall classification accuracy of 84%. In this study, autumn is found to be the most efficient period to acquire monotemporal images for discrimination purposes. At the forest scale, Zhu and Liu [9] investigated the classification of forest types (pine forest, oak forest and mixed forest) from spaceborne image time series (7 Landsat, 7 bands of 30 meters ground sample distance). Their results confirm the importance of phenological information contained in multitemporal data (overall classification accuracy of 90.52%). A similar research on forest-type mapping was performed by means of satellite time series (12 MODIS images, 250 meters resolution). Kempeneers et al. [10] compared the boreal forest-type classification accuracy for each month of a year, and pointed out that the optimal acquisition window is between June and July.

Previous investigations on phenology change, using low aerial imagery, have remained scarce due to prohibitive operational costs. The forerunner study of Key et al. [4] examines a time series of very-high resolution aerial RGB and color infrared photographs, acquired at 9 dates across a single growing season. Based on a multitemporal dataset of 36 cm spatial resolution, the classification of individual tree crowns of 4 deciduous species is investigated in order to determine the optimal acquisition timing. As a result, the imagery of autumn colorfull foliage provided optimal monotemporal classification (overall accuracy of 76%). However, the optimal two-date combination corresponds in this case to spring and midsummer.

Up to now, the use of unmanned aerial systems for precision forestry was focused on the geometric processing of image blocks, in order to deliver an orthophotomosaic and 3D information related to the canopy surface. Although photogrammetry on forested area has always been a challenge, current photogrammetric techniques make it feasible to measure canopy surface height and to generate geometrically reliable orthophotomosaics, based on consumer grade overlapping UAS imagery. Dandois and Ellis [15] have therefore developed a workflow from UAS images to determine the ground elevation during leaf-off conditions, and the canopy surface elevation during leaf-on conditions. The subtraction of soil elevation from the canopy surface elevation resulted in a canopy height model that is used to characterize forest maturity. UAS imagery was also used for classification purposes to perform the early detection of bark beetle attacks in Sitka spruce stands [3] and to discriminate tree and bush species [7].

Owing their low operatial costs and high resolution, drones are measurement devices of prime interest for forest monitoring. Previous investigations on the changes in forest phenology still lack either an imagery covering all phenological events (temporal resolution), or the high spatial resolution required to differentiate individual trees. No dense time series from UAS imagery have yet been used to study the separability variation along the growing period of deciduous trees. Now that UAS technology is mature, the way is open for a real revolution of spatial ecology [1].

### Objectives

Although the promising advances of UAS-based approaches for tree-level forest inventory, a few issues still need to be overcome. First, the determination of the optimal UAS flight configuration is crucial for forested areas. Numerous parameters impact the effective extraction of accurate information from UAS imagery, such as flight altitude, image overlap, resolution, and time windows during the growing season [15]. Moreover, the underlying image processing workflow, based on photogrammetry and image classification techniques, determines the quality of the resulting high resolution thematic forest map [16]. Our research focuses on the determination of the most efficient single-date time windows to identify deciduous tree species. We collected a dense time series of UAS imagery in order to cover the different phenological states. Tree crowns were manually delineated by photointerpretation, and species groups were automatically classified with their spectral response by using supervised Random Forest classifiers. The comparison of classification success for the different UAS surveys makes it possible to draw general guidance regarding the appropriate time windows when UAS acquisition should be performed. In addition, we investigated the efficiency of tree discrimination, based on multitemporal datasets, by comparing the classification accuracy that resulted from two-date and three-date combinations.

Furthermore, we compared two camera systems; a normal camera (RGB camera) and a modified camera for near infra-read acquisition (denoted as CIR for Color infra-read). For every UAS survey, two identical and successive flights were performed, one for each of the RGB and CIR cameras. Accurate co-registration of imagery for these camera systems resulted in multispectral orthophotomosaics (blue, green, red and near infra-red bands) [4].

## Material and Methods

### The study site

The study area is a 130 ha broadleaved forest, located in the municipality of Grand-Leez, Belgium. Stands are mixed and uneven-aged (originating from a coppice). The main species is English oak (*Quercus robur* L.), in mixture with a procession of broadleaved species and a few coniferous trees. The forest habitat conforms to the Atlantic oakwoods, and the soil material is loessal silt loams.

### Unmanned Aerial System survey

#### Description of the UAS and sensors

The Gatewing X100 small UAS (www.gatewing.com) (wingspan: 100 cm, weight: 2.2 kg, cruise speed: 80 km/h, flight height: from 100 m to 750 m, maximum flight duration: 40 minutes, catapult launch and belly landing) is a professional fixed wing UAS dedicated to rapid mapping, and able to cover a relatively large area in a single flight. The flight plans (working area size and location, image overlap, flight altitude, location of take-off and landing points, wind and landing directions) are prepared on the field, prior to the aerial survey, by using a rugged tablet computer (the Ground Control Station). Flights are fully automatic from takeoff to landing and complete stop, although the remote pilot has the possibility to intervene on the flight path whenever there is a risk of accident. The small UAS payload is a compact camera from Ricoh (GR2, GR3 or GR4 still camera—10 megapixel Charged Coupled Device, 6 mm focal length or 28 mm in 35 mm equivalent focal length). We used five different cameras of similar specifications for the 20 flight surveys acquired for the time series. Only two cameras were adapted for near-infrared acquisition by removing the internal hot-mirror filter and adding a blue-light-blocking filter (i.e. a yellow long pass filter) [17]. Shutter speed and camera sensor sensitivity (ISO) are manually selected according to luminosity.

#### The aerial surveys

**Plan of acquisition dates**: Datasets of multitemporal UAS images were acquired for the study area from spring 2011 to autumn 2014. Special attention was given to capture images at each phenology phase. In particular, two temporal windows were considered essential owing the disparity in forest tree phenology: the start and the end of the growing season (early spring and mid-autumn) [4, 5]. Ten acquisition dates covered the period of active growth, from April to November. We performed 2 successive flights for each survey date; one flight with the normal camera, and one flight with the modified camera for near infra-red acquisition. The multitemporal dataset resulted in 20 flights acquired during 10 acquisition dates: 3 surveys were performed in spring, 3 in summer and 4 in autumn. We collected a total of 10058 raw images for the present study over a period of 3 years and a half. Surveys of the time series are numbered and described on Table 1. For the sake of clarity, the surveys are ordered by day and month of acquisition, without taking the year into account. The aerial surveys cover the three seasons of active growth, i.e spring, summer and autumn. Previous investigations have shown that UAS image blocks of leaf-off trees are difficult to handle with photogrammetric processing, in particular because of the lack of identifiable feature points on the images. Flights were therefore performed under leaf-on conditions, except in the case of surveys 1 and 10 for which a significant number of trees are leaf-off. The inter-annual variation in phenology onset was taken into account by characterizing the acquisition dates as Growing Degree Days (GDD, base temperature of 10°C). Data from a meteorological station located not far from the study site (5km away) was analyzed to depict the inter-annual variation of climate. Hence, we noticed that the trend in temperature evolution was well pronounced from year 2011 to 2014: in 2011, the weather was warmer than the average and got gradually more temperate over the 3 following years. In addition, spring of 2013 and 2014 started late and the climate remained cold during the entire growing season.

**Table 1:** Characteristics of the 20 image blocks composing the time series of UAS imagery. 2 successive flights were performed for all 10 acquisition dates; one flight was performed with a visible camera, and one flight was performed with a modified camera for near infra-red acquisition (respectively denoted as RGB and CIR camera). The minimum and maximum altitude, GSD, and the number of images, are emphasized in bold writing.

Survey ID	Date	Season	GDD *	Camera	Altitude [*m*]	GSD **	Overlap [%]	Images	Luminosity changes
1	2012-04-27	spring	64	RGB	225	7,6	77	557	Yes
				CIR	250	8,4	80	574	Yes
2	2011-04-27	spring	178	RGB	**150**	**5**	75	641	
				CIR	**150**	**5**	75	551	Yes
3	2013-05-28	spring	183	RGB	249	8,4	80	481	
				CIR	249	8,4	80	481	
4	2012-06-05	summer	305	RGB	250	8,4	80	635	Yes
				CIR	250	8,4	80	**661**	
5	2013-07-08	summer	491	RGB	**350**	**11,8**	80	320	
				CIR	**350**	**11,8**	80	319	
6	2014-08-21	summer	732	RGB	225	7,6	80	552	
				CIR	225	7,6	80	552	Yes
7	2014-09-18	autumn	897	RGB	225	7,6	80	367	Yes
				CIR	225	7,6	80	**172**	
8	2013-10-01	autumn	1085	RGB	250	8,4	80	473	
				CIR	250	8,4	80	473	Yes
9	2012-10-22	autumn	1409	RGB	225	7,6	75	561	
				CIR	225	7,6	75	560	
10	2013-11-15	autumn	1169	RGB	225	7,6	80	564	Yes
				CIR	225	7,6	80	564	

* Growing Degree Days.

** Ground Sample Distance [*cm*/*pixel*].

**Georeferencing**: To perform an accurate georeferencing of the entire time series, we placed 7 Ground Control Points (GCPs) around the forest prior to survey number 5. The GCPs were materialized by white panels of 50x50cm, supplemented with 4 white strips of 80 cm long on their corners. The coordinates of the GCPs were collected using a Leica GPS1200 GPS in static RTK mode (nominal accuracy of 1 cm in planimetry and 1.5 cm in altimetry) under the Belgian Lambert 1972 projection system.

**Fig 2:**
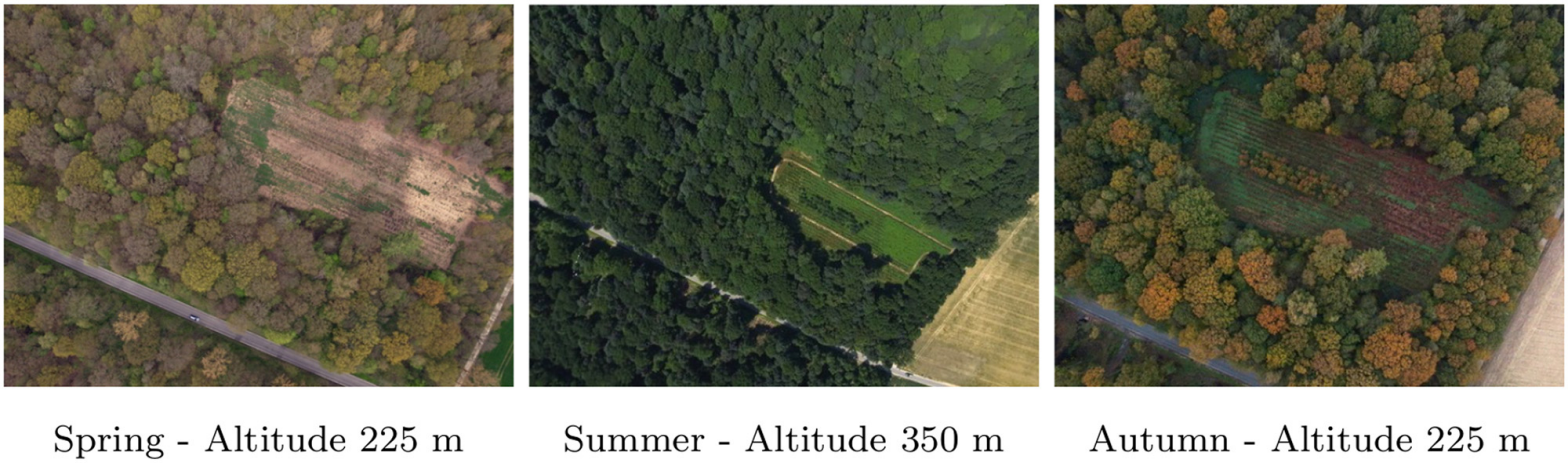
Individual aerial RGB images of a forest opening from survey 1 (spring), 5 (summer) and 10 (autumn).

**Dissimilarities between image blocks**: Image blocks of the time series show some notable dissimilarities. First, as shown on Table 1, the flight configuration varied. More specifically, the flight altitude, which is defined as the altitude above ground level at the take-off location, ranged from 150 to 350 meters. The flight altitude directly impacts the image resolution, the image footprint, and the number of images required to cover the study area at a given image overlap. The choice of the optimal altitude for this survey is thus a trade-off between the resolution and the maximum size of the area scanned during a single flight. The lower the flight altitude, the higher the image resolution and the smaller the scanned area. We considered a flight altitude of 225 meters as optimal via a trial-and-error procedure, taking into account both the study requirements and the size of the forest. Such altitude allows indeed to cover the entire forest in one flight and insures a suitable resolution all at once. In addition, the image footprint was large enough to capture hundreds of tree crowns. The number of objects on an image was thus sufficient to extract the numerous feature points that are at the basis of the entire photogrammetic workflow. The scanning area was defined on the field by drawing freehand the scanning zone on a Google earth map. The survey area thus varied between aerial surveys since the operator did not always define exactly the same scanning zone. The number of images of survey 7-CIR was abnormally low regarding the flight altitude and overlap, due to the malfunctioning of the triggering cable which prevented image shooting at the beginning of the flight. Moreover, changes in luminosity conditions between the different surveys, and between the successive flight lines of a single flight, result in dataset heterogeneity. The image quality of some flights was affected as well by the presence of large shadows casted by trees, even though special attention was given to fly either under cloudy conditions or under solar noon. Still, changes in tree phenology are more responsible than flight configuration, camera type, and luminosity conditions, for the differences between images from various acquisition dates (Fig 2).

#### The photogrammetric workflow

**Fig 3:**
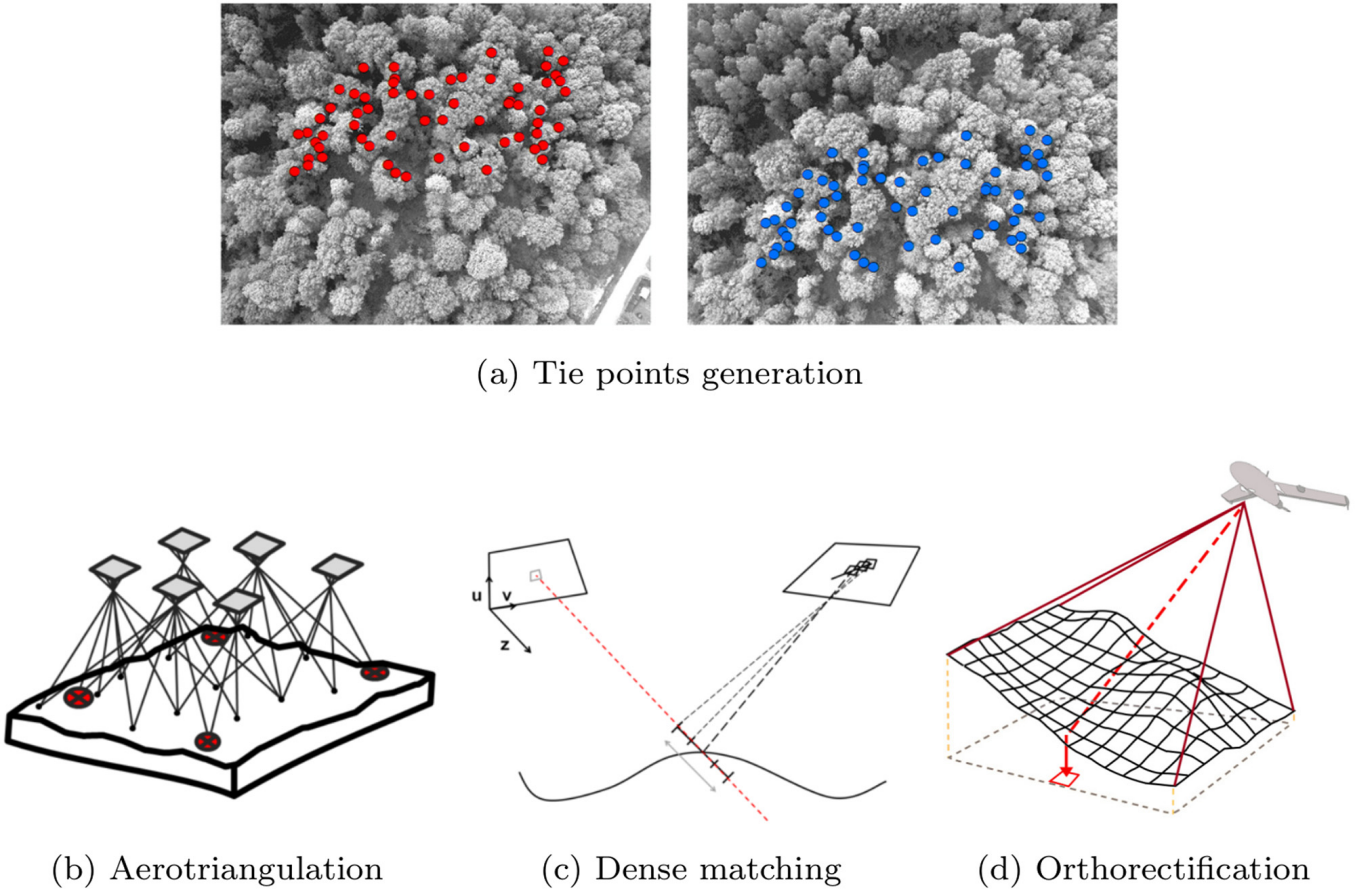
Summary of the photogrammetric workflow. Recent development in software facilitates the automatic processing of hundred of images in order to produce a 3D model (Digital Surface Model) and an orthophotomosaic (see [16] for additional detail).

The 20 image blocks are processed using *Structure from Motion* and photogrammetric techniques, in order to deliver 20 georeferenced orthophotomosaics at a resolution of 20 cm/pixel. Modern aerial photogrammetry starts from an unordered overlapping collection of images, and results in a fine 3D model and a orthophomosaic [15]. The four processing steps, depicted on Fig 3, have recently been implemented efficiently in a large number of software. First, the generation of tie points is subject to an extraction of feature points for each individual image, with a subsequent comparison of image features for overlapping image pairs. When image features, such as tree crowns or road corners, are detected on two images, they are then considered as tie points (illustrated by red and blue dots on Fig 3). Secondly, the orientation of the image blocks (i.e. pose and calibration of the camera) is recovered by aerotriangulation of the tie points, using a Bundle Block Adjustment (BBA) algorithm [18]. Even though the compact cameras used in this study were precalibrated in the laboratory, the internal parameters are still refined during the bundle block adjustment (self-calibrating BBA).

Thereafter, the camera orientations are used to match small pixel windows, thus delivering a fine and dense 3D model of the canopy surface. The dense matching is performed for each successive pair of images, and the resulting stereo-derived depth maps are merged to form a digital surface model over the whole scanned area. Such model is a faithful representation of the relief and is used in the fourth processing step which consists in image orthorectification and mosaicking. The orthorectification procedure removes the geometric distortions caused by image perspective, relief displacement, and lens distortions. Finally, the orthophotomosaic resulting from true orthorectification is suitable for mapping purposes.

The use of photogrammetric algorithms in vegetation areas is quite challenging, due to the numerous vegetation characteristics that hinder image matching: omissions, repetitive texture, and multi-layered and moving objects such as leaves or tree branches [19]. Regarding the generation of tie points, there is a clear lack of feature points from the repetitive texture of tree crowns. Leaf-off conditions or a low image overlap obviously amplified such lack of tie points. A preliminary test showed that these issues have an all the more negative impact if the UAS flies at a low altitude, due to a decrease in the image footprint and, consequently, a decrease in the number of objects on the photograph. In addition, the abrupt vertical changes occurring between tree crowns caused multiple omissions that hinder the dense-matching process. Errors on the Digital Surface Model used for orthorectification have a direct impact on the accuracy of the estimated pixel location [20]. The importance of an accurate canopy surface modelization is thus primordial. Hence, we adjusted the dense matching strategy in order to produce an adequate model of the canopy surface. For this purpose, we used the open source toolbox MICMAC[21] (software revision 1692). We refer the readers to the research of Lisein et al. [16] for additional details on the computation of digital surface model from UAS imagery. All the remaining processing steps (i.e. processing 1, 2 and 4 of Fig 3) are handled with the commercial software Photoscan Professional version 1.0.1 (Agisoft LLC, St. Petersburg, Russia).

Dense matching was performed in image geometry solely for the image block 5-RGB. The resulting Digital Surface Model was used for the orthorectification process of all the other surveys. The selection of the image block 5-RGB for the DSM generation is based on the fact that ground control points are only present during one acquisition date (survey 5). The photogrammetric canopy surface model is generated at a 1:2 initial resolution (GSD of 22 cm).

All the other flights, which did not dispose of GCPs for accurate georeferencing, were co-registered with flight 5-RGB. Flight co-registration is achieved by determining the tie points between the images of one flight and the images of the *master* image block. Image blocks are thus linked with flight 5-RGB through tie points which were mainly located on permanent structures such as roads and houses at the edge of the forest. Aerotriangulation was also performed on images from different surveys, and the resulting image orientation was georeferenced using the GCPs of the *master* image block (5-RGB). Such process ensures a good georeferencing and an accurate co-registration between the flights. Aerotriangulation also takes advantage of the available photogrammetric tools, namely the automatic generation of tie points. Apart for georeferencing, we also used GCPs to achieve constrained bundle block adjustment which supports both tie point and GCPs observations (the optimize tool of Photoscan), in order to remove non-linear distortions that may otherwise taint the photogrammetric models [22]. The georeferencing quality was evaluated for flight 5-RGB by means of the GCPs residuals. For this purpose, GCPs were used one after the other as check points in a leave-one-out approach: 7 additional aerotriangulations, with both tie points and GCPs, were performed in order to obtain a robust measurement quality of the georeferencing. The root-mean-square error of each check point location was then averaged to deliver a global estimation of georeferencing success.

Photoscan parameters were set as followed: maximum 20 000 feature points per image, medium alignment quality, marker accuracy of 0.001 meter, projection accuracy of 0.1 pixel, tie point accuracy of 4 pixels, and mosaic mode for mosaicking. Prior to orthorectification and mosaicking, we manually discarded the images showing a high degree of luminosity change compared to the rest of the image block.

### Field inventory and species phenology

To evaluate the efficiency of UAS imagery for the automatic discrimination of trees, we selected five categories of the predominant forest species: English oak, birches (*Betula pendula* Roth. and *Betula pubescens* Ehrh.), sycamore maple (*Acer pseudoplatanus* L.), common ash (*Fraxinus excelsior* L.) and poplars (two distinct varieties of cultivated *Populus* spp.). The two birch species, silver birch and European white birch, were gathered in the same category due to their phenological similarities. On the other hand, the poplar varieties were brought together as they were difficult to differentiate during field inventory.

The field inventory focuses on mature overstory trees which were clearly identifiable on the time series. We first discarded the area on the forest edge that was not visible on every orthophotomosaic. The resulting study area of 80 ha was completely covered by the multitemporal dataset. Subsequently, in order to achieve photointerpretation on the field, the orthophotomosaics of the time series were loaded on a rugged tablet computer with internal GPS (a Yuma Trimble). Species identification and manual crown delineation on the time series were therefore performed on the field with the mobile field mapping software ArcPad 8.0. Survey operations were carried out in spring 2014. Attention was paid to balance at best the tree sampling for each species and to evenly distribute tree monitoring over the whole study area.

In total, the discrimination of species, and the identification and delineation of tree crowns, were achieved for 577 trees of the 5 different categories: 72 birches, 186 English oaks, 142 sycamore maples, 196 common ashes and 81 poplars.

The benefits of such time series for species classification are based on the capacity to differentiate tree categories through the spectral and phenological differences between English oak, birches, maple, ash and poplars trees. Subtle differences in the timing of recurring biological events may be of crucial importance. Such differences can occur, for instance, in the order of leafing, flowering and fruiting, or the foliage coloring and senescence. The tree phenology varies according to numerous interacting factors, including the ecological conditions, the micro-climate or the vigor status of the tree. The main phenological characteristics of each tree category are reviewed in this paragraph. English oaks at the study site are old and always dominant (>100 years), with well expanded tree crowns. Ashe trees have a late leaf flushing and an early leaf senescence, in comparison to the other tree categories. Moreover, some ash trees are affected by the pathogen *Chalara fraxinea*[23] and therefore show partial crown defoliation during the growing season. Maples and English oaks have a similar timing of vegetation growth, with the onset in May and the senescence in October. However, the English oaks tend to maintain their foliage longer, until November. On the other hand, Birches mainly have small tree crowns and a long growing season (leafing as early as March, and late foliage senescence). The two varieties of poplar present two different phenology patterns since the onset of leaf flushing and leaf fall differs. In addition, one of the poplar varieties has a shorter growing period. Finally, the timing of phenological events is well synchronized for poplars, compared to the other species, as the cloned cultivars of each variety share the same genotype.

### Classification of tree species using Random Forest

#### Computation of metrics

Object-based image analysis was used for the classification surveys of this research [24]. This method was indeed proven superior to pixel-based approaches for very-high spatial resolution. The objects conform to the entire and individual tree crowns which were manually delineated. The spectral response is summarized at the scale of individual tree crowns by computing descriptive statistics from the orthophotomosaics, and denoted as *metrics*. The mean, standard deviation, band ratios and normalized index (one band value over the sum of all the 3 bands) are extracted using the [R] statistical software (version 3.1.0) with the package raster[25]. For false color orthophotomosaics, Green Normalized Difference Vegetation Index (GNDVI), Normalized Difference Vegetation Index and Normalized Green-Red Vegetation Index were computed [8, 26]. Normalized Green-Red Vegetation Index, Normalized Green-Blue Index and Normalized Red-Blue Index were generated from RGB orthophotomosaics. In addition, texture metrics from gray-level co-occurrence matrices were computed by means of the *glcm* package (variance, homogeneity, contrast, dissimilarity, entropy, second moment and correlation).

Prior to the computation of a spectral index on crown areas, we narrowed the crown polygons with a negative buffer of 50 cm in order to remove at best the crown parts which were on the crown border. Only the sunlit parts of the crowns are kept for the metrics computation, as shadows impact negatively the crown spectral response [13, 14]. In this study, we therefore used pixel relative intensity to remove the shadowed areas. Relative intensity was computed as a normalized value of pixel intensity (intensity is the sum of all the three bands), ranging from 0 to 100. We finally identified and discarded the darkest pixel values, considered as shadowed parts, when their relative intensity was below 20%.

#### Random Forest classification

Random Forest (RF) is a supervised and non-parametric method of classification that is widely used in the field of remote sensing (for example [6, 14, 27, 28]). In particular, the RF method has proven its efficiency in managing high dimensional problems [29] (small number of observations but high number of explanatory variables). RF consists in a collection of decision trees. The individual classification trees are trained on a bootstrap sample of observations by randomly selecting a subset of explanatory variables (Random-Input) at each node. Such random trees are subsequently aggregated together in a Random Forest (process referred to as bagging, for ***b**ootstrap **agg**regat**ing***). The bagging of decision trees has the advantage of stabilizing the relation between the exploratory variables and the dependent variable. Furthermore, aggregation of a few RFs together generally results in a stable response of the out of bag error. Out of bag error is a prediction error estimate, based on the out of bag sample. Such sample corresponds to a set of observations which are not used to build the current individual decision trees [29]. This cross-validation estimation is used in this work as an indication of the classification quality, since we focused on model comparison and not on model validation. The package randomForest[30], implemented in the [R] statistical software, is used in this study. In order to avoid class-imbalance problems, the sampling of observations is performed prior to every RF to obtain 50 tree crowns for each category of species.

We addressed the question of the optimal single-date on which the five categories of species are spectrally most separable. We therefore performed a classification scenario at each aerial survey (one survey is made up of two flights, one RGB flight and one CIR flight). A number of 20 RFs (500 decision trees) were generated at every acquisition date. The resulting 20 misclassification out of bag errors (OOB) were averaged for each classification scenario. Moreover, two additional investigations were realized. First, we compared the performance of the RGB camera and CIR camera by undertaking classification scenario solely on the basis of individual flight metrics. Secondly, the added value of multitemporal data in species discrimination was evaluated by classifying tree crowns via survey pairs and trios. The 5 most efficient two-date and three-date combinations are compared and discussed.

**Replication of the classification approach**: The use of the free and open source [R] statistical software enabled easy replication of the classification methods presented in this paper. A simplified dataset of the time series used in this research, as well as the [R] script, are available in S1 Appendix.

## Results and Discussion

### The time series of orthophotomosaics

**Fig 4:**
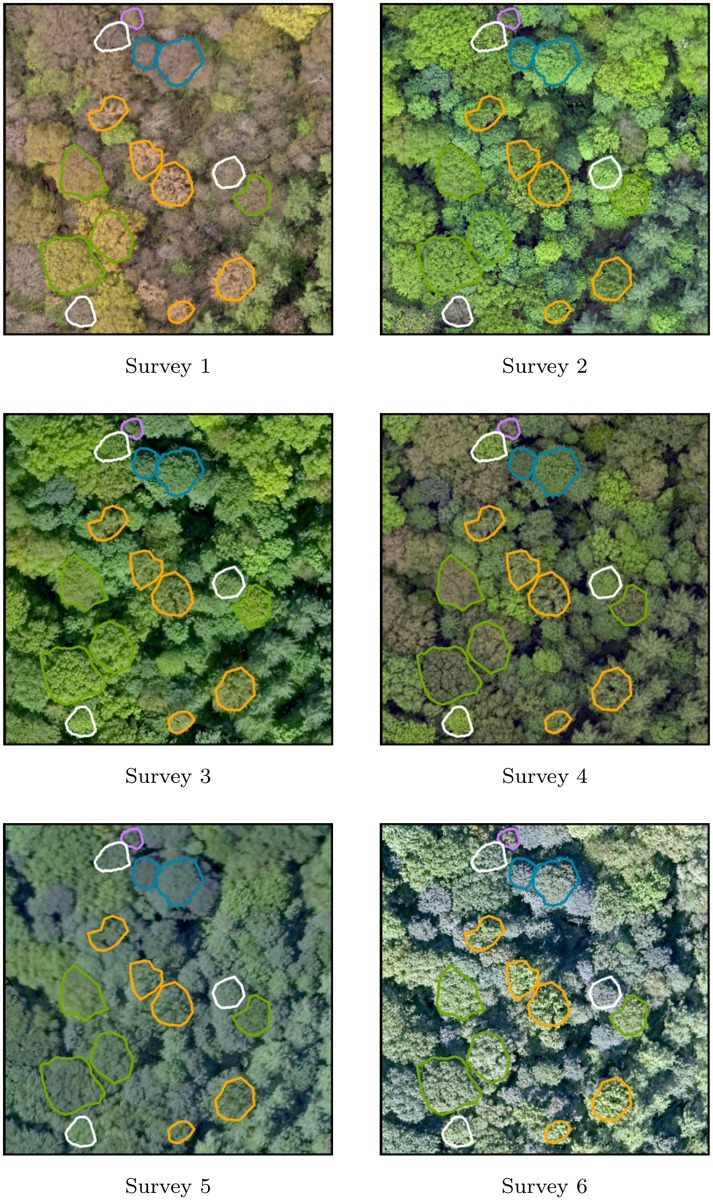
Zoom-in overview of the time series of high resolution forested orthophotomosaics (20cm GSD). The 6 first RGB orthophotomosaics are illustrated. Delineated trees are colored by species; English oak: green—poplars: orange—sycamore maple: blue—common ash: white—birches: purple.

**Fig 5:**
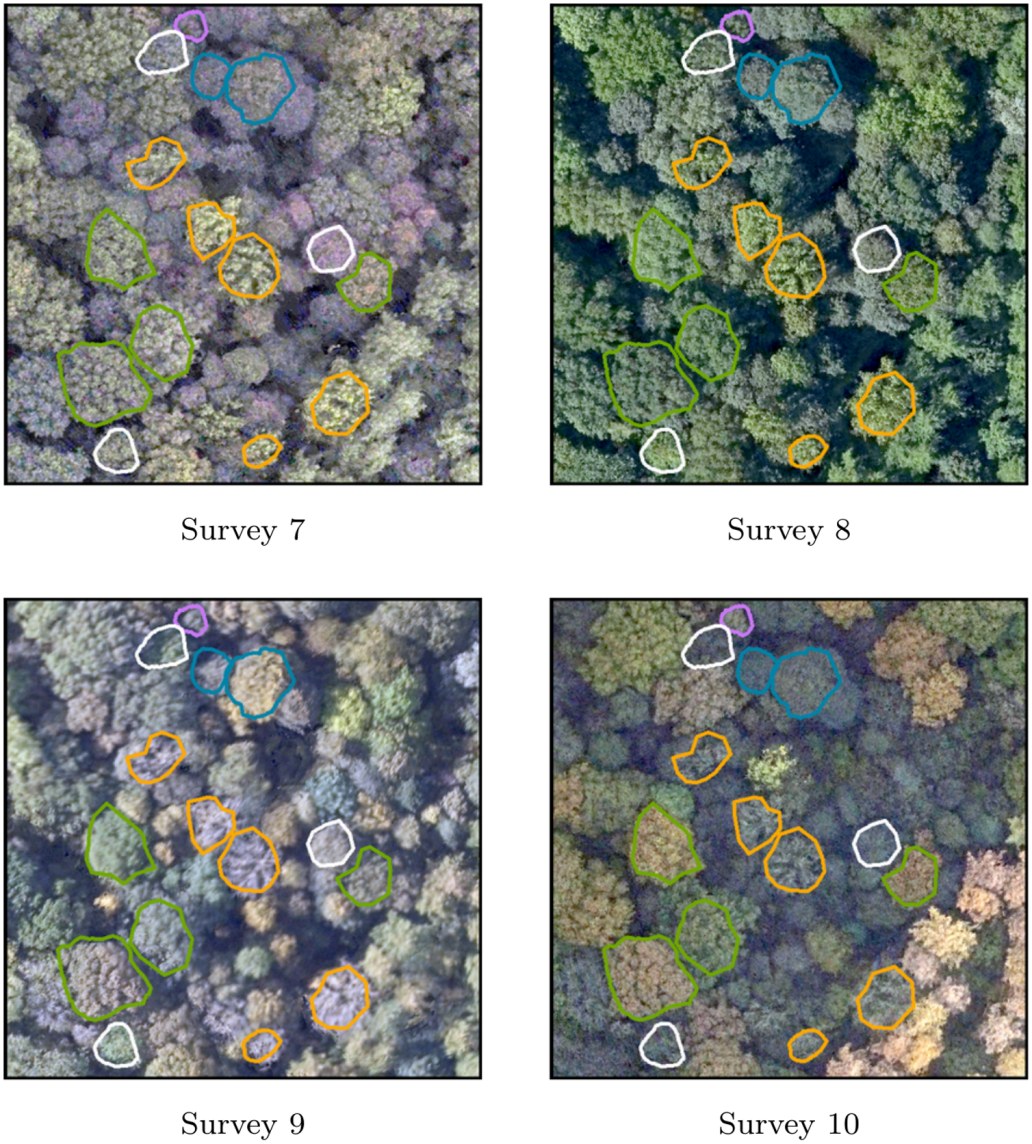
Zoom-in overview of the time series of high resolution forested orthophotomosaics (20cm GSD). The 4 last RGB orthophotomosaics are illustrated. Delineated trees are colored by species; English oak: green—poplars: orange—sycamore maple: blue—common ash: white—birches: purple.

Generation of the time series resulted in a co-registered collection of 10 RGB orthophotomosaics and 10 CIR orthophotomosaics. A visual inspection confirmed that the georeferencing is consistent for every image block. The time series is illustrated on Figs 4 and 5, with superimposed tree crowns colored by species category. Orthophotomosaics of survey 5 and 9 appear locally less sharp than on the other surveys. For survey 5 in particular, such difference is probably due to the higher flight altitude of the survey, resulting in a lower spatial resolution. Owing to the windy condition during the flight, raw images of survey 9 were affected by a smearing effect (motion blur) more pronounced than the other image block. As visible on Figs 4 and 5, the presence of shadows on RGB orthophotomosaics of surveys 3, 6, 8 and 9 prevents the correct visualization of understory trees and forest gaps.

The most striking difference between the orthophotomosaics is the spectral variation among the images of a single block, caused by the rapid changes of luminosity conditions during a flight [31]. Orthophotomosaics affected by such issues are marked on Table 1, and consist of 8 image blocks out of the total 20 image blocks. The spectral information of a given species varies across the study area. As a consequence, the automatic classification of tree species is expected to show weak performances when luminosity conditions change across a single orthophotomosaic.

Although the image blocks are distinct in terms of flight configuration, the photogrammetric workflow removes a major part of the heterogeneity among the surveys. The resolution is the same for each orthophotomosaic of the time series and the georeferencing satisfactory. For flight 5-RGB, the root-mean-square error of residuals on the check point positions is 0.10 meters in altimetry, and 0.35 meters in planimetry. In addition, we used the same digital elevation model for the orthorectification process of all image blocks, and the resulting orthophotomosaics appear error free and present a good level of sharpness. Hence, co-registration of all the surveys was a success.

### Classification of tree species

The classification results for monotemporal surveys, and for a single camera flight, reveal a strong trend (Table 2). Monotemporal acquisitions enable a good discrimination of tree species, with an OOB error ranging from 15.9% to 36.0% across the diverse vegetation periods. The best surveys are shown to be the survey 3, 4 and 2, which were all achieved at the end of the leaf-flushing event. Accordingly, spring and early summer are highlighted as the optimal time windows for the discrimination of broadleaved trees. On the other hand, surveys realized after June do not reach an out of bag error below 30%.

**Table 2:** Classification error for each flight and for each survey (combination of CIR and RGB flights). Surveys in spring and early summer gave the best results and the RGB camera clearly outperforms the color infra-red camera.

Survey	Date	Camera	Out of bag error [%]					
			overall	birches	English oak	sycamore maple	common ash	poplars
		RGB	33.8					
1	2012-04-27	CIR	39.3					
		RGB+CIR	26.7	12	27	63	28	**5**
		RGB	29.4					
2	2011-04-27	CIR	32.3					
		RGB+CIR	**23.4**	10	35	30	32	10
		RGB	17.8					
3	2013-05-28	CIR	38.4					
		RGB+CIR	**15.9**	**6**	**13**	**25**	29	**5**
		RGB	22.1					
4	2012-06-05	CIR	40.6					
		RGB+CIR	**18.1**	11	25	28	**17**	10
		RGB	31.3					
5	2013-07-08	CIR	51.4					
		RGB+CIR	30.1	23	32	31	38	28
		RGB	34.8					
6	2014-08-21	CIR	61.5					
		RGB+CIR	34.8	18	50	48	44	15
		RGB	38.9					
7	2014-09-18	CIR	47.5					
		RGB+CIR	33.6	15	34	57	53	10
		RGB	32.7					
8	2013-10-01	CIR	53.7					
		RGB+CIR	30.5	22	35	33	52	11
		RGB	43.1					
9	2012-10-22	CIR	49					
		RGB+CIR	36	40	47	43	37	12
		RGB	36.7					
10	2013-11-15	CIR	43.5					
		RGB+CIR	31	21	22	63	32	17
average				18	32	42	36	12

Comparison of RGB and CIR camera efficiency to discriminate species clearly shows that the RGB camera outperforms the CIR camera. Even though the differences in classification accuracy varies a lot across the time series, the CIR orthophotomosaics always presents an overall out of bag error which exceeds the performances of the RGB orthophotomosaics by 14% in average. The average gain of a combined use of RGB and CIR orthophotomosaics accounts only for 4%, over the use of RGB orthophotomosaics alone.

The added value of multitemporal datasets is clearly significant: the lowest out of bag error for a two-date classification is 11.3% (Table 3). For the three-date combinations, the accuracy of the classification increases, with a classification error of 8.8% for the optimal combinations. Every date combination, illustrated in Table 3, involves the survey number 3 (2013-05-28) which was demonstrated as the optimal single-date acquisition. Survey 4 is also repeatedly involved in date combinations. Indeed, the five best three-date combinations all consist of both surveys 3 and 4. Moreover, survey 4 which occurred in early summer (2012-06-05) is identified as the second optimal monotemporal survey (Table 2). In addition, the overall and by-class classification error is very similar for each combination, although the two-date and three-date combinations consist of various seasons. The mid-summer surveys (number 5 and 6) are the only ones that do not appear among the five preferential two-date and three-date combinations. Finally, all the remaining surveys are involved at least once in the various date combinations (Table 3).

**Table 3:** Added value of multitemporal datasets for species discrimination. The 5 best two-date combinations and the 5 best three-date combinations. Survey 3 (**2013-05-28**, highlighted in bold) was present in all the combinations, and survey 4 (*2012-06-05*, in italic writing) was involved in all the three-date combinations.

		Out of bag error [%]					
Dates combination	seasons	overall	birches	English oak	sycamore maple	common ash	poplars
**2013-05-28** and 2012-04-27	early spring/spring	11.3	4	9	26	16	1
**2013-05-28** and *2012-06-05*	spring/early summer	11.3	1	11	26	16	2
**2013-05-28** and 2014-09-18	spring/autumn	11.3	1	11	24	19	1
**2013-05-28** and 2013-11-15	spring/autumn	11.6	4	9	23	20	2
**2013-05-28** and 2011-04-27	early spring/spring	11.4	4	10	24	17	3
average			3	10	25	18	2
**2013-05-28**, *2012-06-05*, 2013-10-01	spring/early summer/autumn	8.8	0	7	23	13	1
**2013-05-28**, *2012-06-05*, 2013-11-15	spring/early summer/autumn	8.8	1	8	21	13	1
**2013-05-28**, *2012-06-05*, 2012-04-27	early spring/spring/early summer	9	0	8	23	13	0
**2013-05-28**, *2012-06-05*, 2012-10-22	spring/early summer/autumn	9.1	1	9	20	14	1
**2013-05-28**, *2012-06-05*, 2014-09-18	spring/early summer/autumn	9.2	0	9	24	13	0
average			1	8	22	13	1

Focus must be given to the different groups of species showing variable levels of separability. First, poplars and birches trees are the most spectrally separable categories. By means of three-date multitemporal imagery, these groups of species are indeed discriminated with a 100% accuracy (Table 3). Such result is remarkable, given that the poplar category consists of two cultivars, both caracterized by different phenology timings.

Sycamore maple is the most difficult species to discriminate, in accordance with the results on field maple of Hill et al. [5]. Similarly, common ash is hard to identify properly, since this tree species is often confused with sycamore maple (confusion matrix is provided in S1 Appendix). On the other hand, English oaks are intermediate in terms of separability. Finally, the confusion in species identification is most prononced between maple and ash. But the spectral response of English oaks is also overlapping the spectral response of maple trees and, to a lesser extent, the one of ash trees.

On the basis of the survey rankings, we selected the most efficient orthophotomosaics for an additional and thorough visual inspection of the tree crowns. Orthophotomosaics of surveys 3 and 4 are far from being the most colorful in the time series. Visually, the more contrasted surveys are the ones performed at the very beginning of the vegetation growth period, and depicting a colorful autumn foliage. By contrast, the spectral response of leaf-on trees during late spring and early summer is more homogenous, since all the individuals were green at these seasons. The phenology status within every category of species is synchronized. In addition, the variability of phenology and spectral response within each group of species is less pronounced at the end of complete leafing, compared to autumn or early spring. Such results underline the importance of intra-species variations in phenology, for species discrimination. The disparity in forest tree phenology is at the maximum during early spring and late autumn. By contrast, the disparity in phenology is at the lowest during summer. The end of leaf flushing, highlighted as the optimal monotemporal time windows, minimizes the spectral variation within tree species groups and, at the same time, maximizes the phenologic differences between species.

Some of the causes of phenology variability within a category of species are related to the forest sylviculture and the history of the study site. The complex vertical structure of the stands generates a micro-climate that can slightly impact the phenology timing. For instance, edges and gaps are numerous in the forest, and trees at these locations are less influenced by the below-canopy micro-climate. The micro-climate is thus more variable in the study site than in even-aged stands. In addition, an important number of common ash trees suffer from ash dieback disease (*Chalara fraxinea*). The visual aspect of the infected tree crowns is therefore affected [32]. Defoliation is particularly visible on survey 2, on which tree crowns appear porous due to the alternation of green branches and necrotic areas.

Another variation in intra-species phenology consists of state of maturity of the trees. English oaks in our surveys are mainly old tree crowns, but a few are young individuals. These young trees present a smaller crown, as well as a different phenology timing and a distinct spectral response.

Sycamore maples, for their part, seem to naturally present a large diversity of spectral responses. Such diversity is particularly visible in autumn, when the yellow tints of maple crowns are mixed with the brown color of fruits, and with the red, green and dark green color of foliage.

## Conclusions and Perspectives

### UAS operations and orthophotomosaic generation

A dense time series of high resolution images was collected successfully across the vegetation period by using a small UAS. The tracking of phenological events such as bud burst, leaf flushing, autumn coloring and leaf fall, was made possible at an affordable price. Using the Gatewing X100, we determined that the appropriate flight altitude for tree-based UAS inventory was 225 meters above ground level. This altitude allows to cover the entire forest estate in one single flight with a good image resolution. In total, 10 surveys were performed at different dates, with each survey consisting of 2 UAS flights: a first flight using a visible camera and a second flight using a color infra-red camera. The ease of use of the small unmanned aerial vehicle used in this study allowed to collect more than 10058 raw aerial images (decimetric resolution), depicting the subtle forest changes. The continuous growing of computation power, as well as the recent advances in modern photogrammetric algorithms, have gradually overcome the technological limitations for the handling of thousands of low-oblique UAS images. By assembling image blocks from an individual flight, we obtained a suitable orthophotomosaic for measurements. Overall, photogrammetric processing allows the removal of most of the disparity between image blocks: images shooted at an altitude of either 350 meters or 150 meters resulted in an orthophotomosaic of 20 cm resolution, regardless of the image perspective. However, improvements are still to be done, particularly in the mitigation of spectral variations among the images of a block, which are caused by rapid changes in luminosity conditions. Radiometric equalization techniques are devoted to the reduction of such spectral variations, although these algorithms still need a better fitting to the characteristics of UAS imagery.

Co-registration of the whole time series, by using one image block as a master and by georeferencing with Ground Control Points, ensures a good consistency between different flights. An alignment of image blocks was achieved by finding tie points between multitemporal image datasets, using structure from motion techniques. An accurate georeferencing was thus performed by taking full advantage of photogrammetric techniques. Moreover, image dense matching, which is an essential process prior to the orthorectification, was solely carried on the master image block for the canopy surface modelization. The resulting Digital Surface Model was used for the whole time series, thus saving the computation time that would have been required to generate the relief for each individual image block.

### Classification of species groups

Individual tree crowns were manually delineated, and a supervised classification of deciduous tree species was performed with Random Forests. Across the study site of 80 ha, we carried out surveys over 577 dominant tree crowns of 5 different groups of species. The optimal phenology state for the discrimination of species was demonstrated to be the end of leaf flush. The intra-species phenology is well synchronized during this optimal time windows ranging from late spring to early summer. At the same time, this time windows still present differences between species, whereas disparities fades away during summer. Hence, we conclude that the intra-species spectral variation is of prime interest for species discrimination. These results contrast with the conclusions of some previous scientific studies, in which the best monotemporal time windows was the one with the highest disparity in forest tree phenology (early spring and late autumn). Although no clear statement has previously been made regarding the optimal time windows, a number of studies have highlighted the autumn season as the optimal single-date time window for species classification [4, 5, 33]. However, these research are lacking imagery covering all the phenological events. The results presented in this article are in line with the study of Kempeneers et al. [10] where a dense time series was used as well, although there are strong differences in terms of image resolution and classification (forest-type mapping versus tree-level species classification).

The use of multitemporal datasets improved considerably the overall classification accuracy. The optimal survey number 3 (end of May) was present on every date combinations, while no recurring combination of seasons stands out in the best two-date and three-date combinations. Such consistency confirmed the importance of determining an optimal time window for species discrimination.

Although the infra-red band is well known to be effective in vegetation mapping, the color infra-red camera was clearly less efficient than the RGB camera. All three bands of the consumer grade CIR camera were indeed sensitive to the near infra-red light, due to an internal modification of the camera for near infra-red acquisition. Redundant band sensitivity led to a spectral overlap between the bands and finally affected the performance of the camera for the discrimination of species. However, the combination of CIR and RGB together proved to be interesting for species discrimination by enhancing the classification accuracy. In addition, the simultaneous acquisitions of RGB and CIR image blocks increased image overlap, thus influencing positively photogrammetric processing. The CIR camera should thus be used only in combination with the RGB camera. Nonetheless, the costs related to an additional flight for CIR image acquisitions make it interesting only for specific case of studies.

The spectral variation within species groups was the main factor affecting the classification of species. As a consequence, all the variations in tree phenology within a group of species had a negative impact on the overall classification performance. For instance, the ash dieback disease had an important effect on the ash canopy spectral response, and was thus a clear issue for ash tree classification. The multi-storied forest of Grand-Leez is favorable for a variety of ecological conditions and micro-climate. Disparity in intra-species phenology is thus greater than in even-aged and mono-specific stands, making species discrimination more challenging. Better results are expected on even-aged stands with the implemented methodology. In addition, investigating the phenological variability for a given species could become a means to study the effect of ecological conditions. Indeed, an even-aged and mono-specific stand which is in good health will show phenological differences mainly under changes in genetics or ecological conditions.

The various categories of species showed diverse degrees of separability. Birches and poplars were easily classified, whereas sycamore maple showed such a variability in phenology that distinguishing it from ash or English oak remained difficult. Even inside a single sycamore maple crowns, the branches showed various colors, especially during the leaf fall and colouring stage. Although the classification object in this study was the individual crown, no test regarding the appropriate scale of investigation was performed. The sycamore classification could therefore be carried out more efficiently by focusing on objects of smaller size, such as branch level. Future studies evaluating the effect of the object size from high resolution images on species discrimination should be investigated, in order to optimize the classification of deciduous tree crowns.

### Perspectives

Monotemporal UAS imagery was considered as a promising tool for the determination of forest species, with a global classification error of 16% at the optimal time window. Biodiversity monitoring and the assessment of forest resources could greatly benefit from such accurate and automatic mapping of species at tree level. The aim of this study was to define the optimal single-date time window for species discrimination. The fact remains that the costs of UAS acquisition are not prohibitive, making multitemporal surveys largely affordable in the context of scientific research. The value of multitemporal imagery was clearly confirmed in this study.

The chosen methodology can be considered as one of the many ways to use UAS imagery for forestry purposes. Numerous scientific topics can be investigated by means of such cost-effective tool. The determination of tree species from spectral information is part of the various informations that can be provided by UAS imagery. Tree height and crown size or shape can also be efficiently measured from drone imagery. The volume of tree biomass and wood can finally be derived from these measurements through the use of allometric models. UAS are clearly cost-effective and non intrusive methods, and the recent advances in computer power and image software make it now possible to handle thousands of aerial images.

The time series of orthophotomosaics will be used in a new study to establish guidelines for photointerpreters for the classification of forest species. Orthophotomosaics shall be used as well as raw individual aerial images. Such images are indeed sharper than orthophotomosaics, with a low oblique view which can help the manual determination of trees. Since each image is georeferenced, switching between image and terrain geometry is not an issue.

## Supporting Information

S1 Appendix Replication data for the discrimination of tree species based on UAS imagery. The supplementary material includes the 10 Red-Green-Blue orthophotomosaics of the time series, the delineated tree crowns, a tutorial and the [R] source code required to replicate the classification approach.
(PDF)
