# Supplementary material for: Discrimination of Deciduous Tree Species from Time Series of Unmanned Aerial System Imagery
Source: PLoS One. 2015 Nov 24;10(11):e0141006. doi: 10.1371/journal.pone.0141006 (PMC4657984; doi:10.1371/journal.pone.0141006)
Supplement: S1 Appendix — The supplementary material includes the 10 Red-Green-Blue orthophotomosaics of the time series, the delineated tree crowns, a tutorial and the [R] source code required to replicate the classification approach. (PDF) [file pone.0141006.s001.pdf]

Supporting Information 1:

Replication data for the discrimination of tree species based on UAS imagery.

Description of the Dataset

Introduction This appendix aims at reproducing the results presented in the paper entitled "Discrimination of deciduous tree species from time series of unmanned aerial system imagery".

Research question What is the best time window in the growing season to acheive an optimal discrimination of tree species based on Unmanned Aerial System Imagery.

Data The supplementary material contains the 10 Red-Green-Blue orthophotomosaics of the time series (TIFF raster files numbered 1RGB.tif, 2RGB.tif,... 10RGB.tif). In addition, a shapefile of the manually delineated tree crowns is provided (TreeCrown.shp in ESRI format). A total of 577 trees from 5 different species groups is surveyed. This document contains both the [R] source code and some explanations concerning the applied processing chains in order to answer the research question. The source code is added in a separate [R] document (file timeSeries_GL_Appendix.R). Finally, as the metrics computation processing is time-consuming, only the result of this process is provided to the user (file metrics_species_TS.txt).

Material and Method Crowns of known tree species were manually delineated on the time series. The subsequent discrimination of these crowns by means of Random Forest classification enabled the selection of the most appropriate time series orthophotomosaic.

Differences between the supplementary material and the data used in the original research In order to be as self-explanatory as possible, the dataset presented in this supplementary material has been simplified. Firstly, only the 10 RGB orthophotomosaics of the time series are presented. The 10 color-infrared orthophotomosaics are not used, as the RGB camera was clearly shown to outperform the color-infrared camera. Secondly, the high resolution of the orthophotomosaics has been resampled in order to decrease the volume of the dataset. Orthophotomosaics have a resolution of

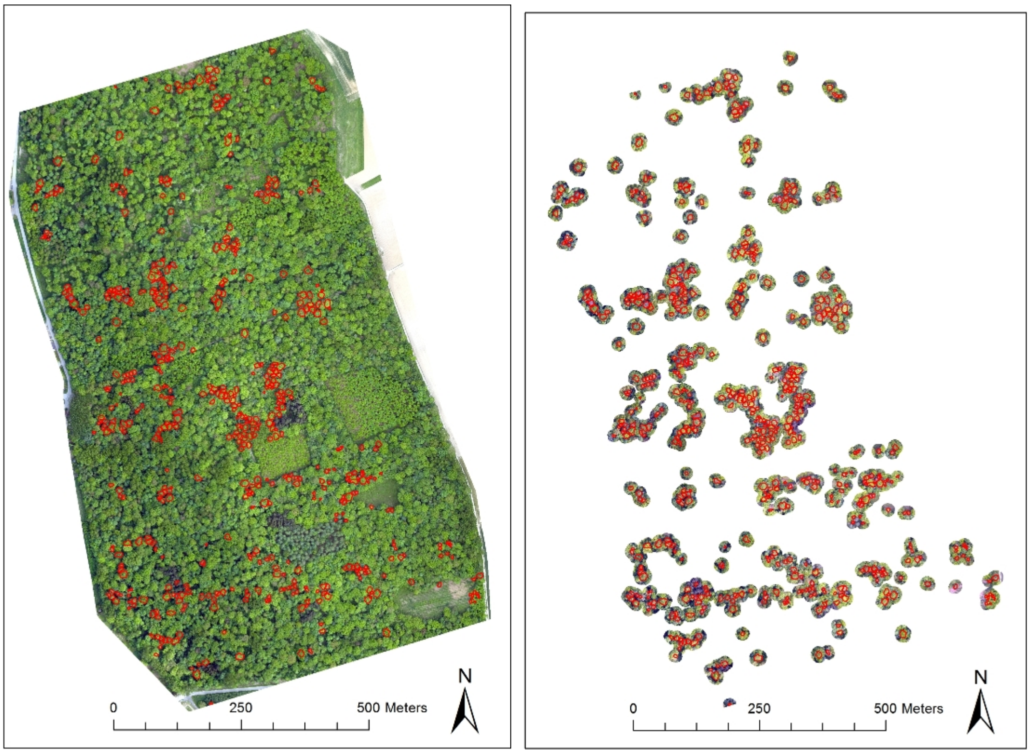

Initial orthophotomosaic: 20cm/pixel   Simplified orthophotomosaic: 40cm/pixel

Figure 1: The time series of this supplementary material has been simplified in order to decrease both size of the data and time of the processing. Spatial resolution of originally 20 cm/pixel (left) has been resampled to a resolution of 40cm/pixel (right) and area without surveyed tree crowns (depicted in red on maps) have been discarded.

20cm in the original research, and a resolution of 40cm in this dataset. In addition, the area of the orthophotomosaics which is not used in the analysis has been discarded (see figure 1). Finally, the training of the diverse classification scenarios slightly differs from the one used in the original paper: only one random forest, instead of 20, is trained for each classification scenario. However, all these differences do not affect qualitatively of the results reached in this study. The optimal time window for the discrimination of this group of species is the end of leaf-flushing (late spring).

Description of the processing chain

The complete workflow is split in 4 steps:

1. Load the data in [R] environment.
2. Display a zoomed view of the time series on a tree crown for the purpose of visual inspection.
3. Compute metrics for each individual crown (typical approach of object-based image analysis).
4. Use Random Forest to build a classifier and compare the classification accuracy of different scenarios:
   (a) classification based on one date (one single orthophotomosaic).
   (b) classification based on a combination of 2 dates (pair of orthophotomosaics).

The implementation of these four processing steps is presented in the following code listings. Relevant results are presented in tables 1 and 2, and in figures 2 and 3.

The processing step "Compute metrics for each individual crown" is time consuming and is not compulsory as the metrics are provided with the supplementary material.

Step 0: load required packages

```
# Goal: compare 10 Unmanned Aerial System flights performed accros the
    growing season in order to determine which phenology stade is the most
    appropriate for forest species discrimination
# 0) load required packages

require(raster)
require(rgeos)
require(randomForest)
require(rgdal)
require(glcm)
require(doParallel)

# set working directory
setwd("Your/Directory")
```

Step 0: load required packages

Step 1: load the data

```
   # ————————————————————————
 2 # 1) load the data

 4 # load the shapefile of delineated tree crown
   trees.shp <- readShapePoly("TreeCrown")
 6 summary(trees.shp@data$SPECIES)

 8 # load the 10 orthophotomosaics (10 acquisition dates, 10 red-green-blue orthophotomosaics) :
   ortho.names <-c("1RGB","2RGB","3RGB","4RGB","5RGB","6RGB","7RGB","8RGB","9RGB","10RGB")
10 # the time series of raster is handled in a list [R] object
   time.series <- sapply(paste(ortho.names, ".tif", sep=""), brick)
```

Step 1: load the data

birches	72
common ash	96
English oak	186
poplars	81
sycamore maple	142
total	577

Table 1: Result of line 6, step 1. Number of tree species observations used in this research.

Step 2: Display a zoomed view of the time series on a tree crown

```
# ————————————————————————
# 2) Display a tree crown

# crop the time series on crown number 260 (a large English oak)
crown.row <- 260
ts_crown <- sapply(time.series, crop, y=gBuffer(trees.shp[crown.row,],width=7.5))
# create a graph illustrating the evolution of the phenology of this tree across the time series
png(paste("illustration_TS_crown",crown.row,".png",sep=""), width=1000, height=400)
par(mfrow=c(2,5))
# loop on ortho in the time series
for (ortho.num in c(1:length(ts_crown))){
  plotRGB(ts_crown[[ortho.num]],stretch="lin")
  box(col="white", lwd=7)
}
# close graphic device
dev.off()
```

Step 2: Display a zoomed view of the time series on a tree crown

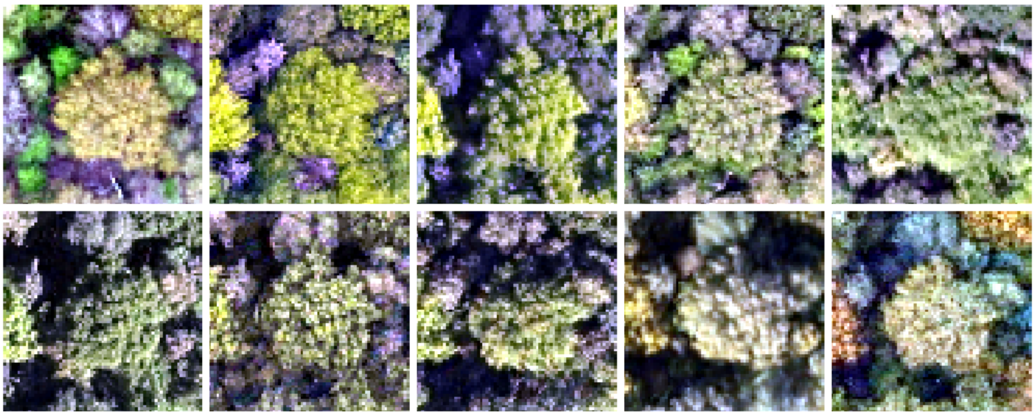

Figure 2: Result of lines 8-16, step 2. Evolution of the aspect of a large English oak across the time series

Step 3: Compute metrics for each individual crown

```
# ----------------------------------------
# 3) Compute metrics for each individual crown. Quite slow (> 1hour). One
    can skip this step as the metrics are available with the dataset (go
    directly to step 4)

registerDoParallel(cl = detectCores()-2) #choose how many cores is used
    during parallel computations

# prepare the list of names for all the metrics.
metrics.names <- c("t.MEAN","t.MEAN.sd", "t.HOM", "t.HOM.sd",  "t.VAR", "t.
    VAR.sd",  "t.CON", "t.CON.sd", "t.DIS", "t.DIS.sd","t.ENT.sd","t.ENT", "
    t.SEC", "t.SEC.sd","R.m", "R.sd","G.m","G.sd", "B.m","B.sd","r.G.R","r.B
    .R", "r.G.B", "n.R","n.G","n.B", "NDGRI.m","NDGRI.sd", "NDGBI.m","NDGBI.
    sd","NDRBI.m","NDRBI.sd")
all.metrics <- sort(levels(interaction(ortho.names, metrics.names)))

# loop on every polygon (crown) of the shapefile
for (crown.row in c(1:nrow(trees.shp))) {

      # select the crown on which metrics will be computed
      crown <- trees.shp[crown.row,]
      # clip the time series around this crown
      ts_crown <- sapply(time.series, crop, y=crown)

      # loop on every orthophotomosaic of the time series and compute the
          metrics. The computation is performed in parallel. The foreach
          loop return a vector of metrics which are subsequently saved.
      metrics.crown <- foreach (ortho.num = 1:length(ts_crown), .packages =
          c("raster","glcm"), .combine="c") %dopar% {

          # remove pixels ouside the crown polygon
          ortho <- mask(ts_crown[[ortho.num]], crown)
          # compute intensity (R+G+B) in order to remove shadows present on
              the crown
          I <- sum(ortho)
          # relative intensity
          Ir <-  100*(I - I@data@min)/(I@data@max - I@data@min)
```

```
# computation of texture metrics on the relative intensity
tx <- glcm(Ir,window = c(3,3))
# summarize the texture at the crown level (mean and standard deviation)
t.MEAN <- mean(values(tx$glcm_mean), na.rm=T)
t.MEAN.sd <-sd(values(tx$glcm_mean), na.rm=T)
t.HOM <- mean(values(tx$glcm_homogeneity), na.rm=T)
t.HOM.sd <- sd(values(tx$glcm_homogeneity), na.rm=T)
t.VAR <- mean(values(tx$glcm_variance), na.rm=T)
t.VAR.sd <- sd(values(tx$glcm_variance), na.rm=T)
t.CON <- mean(values(tx$glcm_contrast), na.rm=T)
t.CON.sd <- sd(values(tx$glcm_contrast), na.rm=T)
t.DIS <- mean(values(tx$glcm_dissimilarity), na.rm=T)
t.DIS.sd <- sd(values(tx$glcm_dissimilarity), na.rm=T)
t.ENT.sd <- sd(values(tx$glcm_entropy), na.rm=T)
t.ENT <- mean(values(tx$glcm_entropy), na.rm=T)
t.ENT.sd <- sd(values(tx$glcm_entropy), na.rm=T)
t.SEC <- mean(values(tx$glcm_dissimilarity), na.rm=T)
t.SEC.sd <- sd(values(tx$glcm_dissimilarity), na.rm=T)

# remove shadow zone if there are some
mask_shadow <- cut(Ir, breaks=c(20,100))
ortho <- mask(ortho, mask_shadow)
# isolate the 3 bands. band 1 : red. band 2: green. band 3 : blue.
b1 <- subset(ortho, 1)
b2 <- subset(ortho, 2)
b3 <- subset(ortho, 3)
b123 <- b1+b2+b3
# compute mean and standard deviation for each band
m1 <- mean(values(b1), na.rm=T)
sd1 <- sd(values(b1), na.rm=T)
m2 <- mean(values(b2), na.rm=T)
sd2 <- sd(values(b2), na.rm=T)
m3 <- mean(values(b3), na.rm=T)
sd3 <- sd(values(b3), na.rm=T)
# simple ratio
ratio1 <- mean(values(b2/(b3+1)), na.rm=T)
ratio2 <- mean(values(b1/(b3+1)), na.rm=T)
ratio3 <- mean(values(b2/(b1+1)), na.rm=T)
# red, green and blue transformation
t1 <- mean(values(b1/b123), na.rm=T)
t2 <- mean(values(b2/b123), na.rm=T)
t3 <- mean(values(b3/b123), na.rm=T)
# NDGRI normalized difference green red index
r.NDGRI <- (b2-b1)/(b2+b1)
NDGRI <- mean(values(r.NDGRI), na.rm=T)
NDGRI.sd <- sd(values(r.NDGRI), na.rm=T)
# NDGBI normalized difference green blue index
r.NDGBI <- (b2-b3)/(b2+b3)
NDGBI <- mean(values(r.NDGBI), na.rm=T)
NDGBI.sd <- sd(values(r.NDGBI), na.rm=T)
# NDRBI normalized difference red blue index
r.NDRBI <- (b3-b1)/(b3+b1)
NDRBI <- mean(values(r.NDRBI), na.rm=T)
NDRBI.sd <- sd(values(r.NDRBI), na.rm=T)
# compile the metrics in a vector which is returned by the foreach parallel loop
metrics <- c(t.MEAN,t.MEAN.sd, t.HOM, t.HOM.sd, t.VAR, t.VAR.sd, t.CON, t.CON.sd, t.DIS, t.DIS.sd,t.ENT.sd, t.ENT, t.SEC, t.SEC.sd,m1,sd1,m2,sd2,m3,sd3,ratio1,ratio2,ratio3, t1,t2,t3, NDGRI,NDGRI.sd, NDGBI,NDGBI.sd,NDRBI,NDRBI.sd)
# determine the names of the current ortho
```

```
            ortho.name <- ortho.names[ortho.num]
            # give the correct names at each element of the vector
            names(metrics) <- paste(ortho.name,metrics.names,sep=".")
            # return the results
            metrics
            # next orthophotomosaic
            }
        # save the metrics in the dataframe of the tree crown shapefile
        for (metric in all.metrics){
        trees.shp@data[crown.row,metric] <- metrics.crown[metric]
        }
# goes on with the next tree crown
cat(paste("End of metric computation for crown number ", crown.row, ", ",
    nrow(trees.shp) ," crowns in total -------------- \n"))
}
# save the result in a txt table
write.table(as.data.frame(trees.shp@data), file="metrics_species_TS.txt",
    sep="\t")
```

Step 3: Compute metrics for each individual crown

Step 4A: classification based on one orthophotomocaic

```
# ------------------------------------------
# 4) use of Random Forest for building a classifier and comparing
    classification accuracy of different scenarios

# read file with species and metrics
crowns.metrics <- read.table("metrics_species_TS.txt", sep="\t")

# 4A) classification based on single date orthophotomosaic

# creation of a matrix for storing result of the different classification
    scenarios
classif_Monotemporal <- matrix(0,ncol=6, nrow=length(ortho.names), dimnames=
    list(ortho.names, c("overall out of bag error", "birches","common ash","
    English oak","poplars","sycamore maple")))

# run one random forest per acquisition date
for (ortho.num in c(1:length(ortho.names)) ){
  # select the metrics related to the ortho by means of regular expression
  regex <- ortho.names[ortho.num]
  selected.metrics <- crowns.metrics[, grep(regex, names(crowns.metrics))]
  # train the random forest
  rf <- randomForest(crowns.metrics$SPECIES~ ., data=na.roughfix(selected.
      metrics), ntrees.shp=500,replace=T,na.action=na.omit)
  # save the random forest accuracy result in the result table
  classif_Monotemporal[ortho.num, 2:6] <- round(100*rf$confusion[1:5,6], 0)
  classif_Monotemporal[ortho.num,1] <- round(100*mean(rf$confusion[,6]), 1)
  cat(paste("End of training Random Forest classifier for ortho ", regex, "
      ------------------------ \n"))
}


# plot of the trend of classification accuracy through the growing season.
    The trend show that end of flushing is the optimal time windows for
    species discrimination
matplot(classif_Monotemporal, type = c("b"),pch=20,lwd=c(5, rep(0.5,5)),col
    =c(2,3:7), lty=c(1),xlab="orthophotomosaic in the time series", ylab="
    Species classification error [%]",main="Evolution of species
    classification throught the growing season",xaxt="n")
```

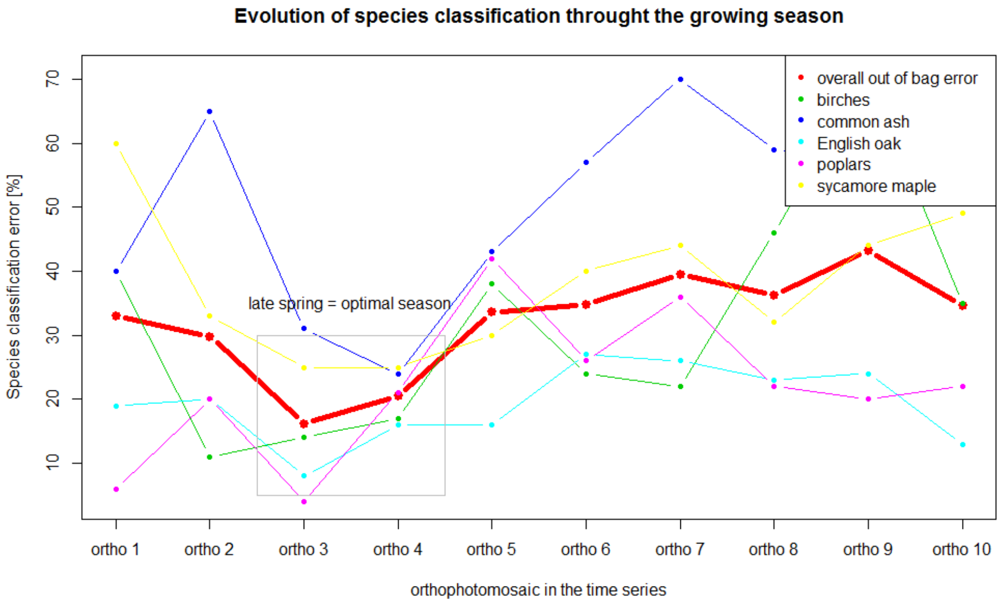

Figure 3: Result of lines 27-31, step 4A. Evolution of the classification accuracy across the time series. Best performances are reached for late spring (orthophotomosaics 3 and 4).

```
   rect(xleft=2.5,ybottom=5,xright=4.5,ytop=30, border="grey")
29 text(x=3.5,y=35,label="late spring = optimal season")
   axis(side=1, at=c(1:10), labels=paste("ortho",c(1:10)))
31 legend("topright", legend = colnames(classif_Monotemporal), col=c(2,3:7),
     pch=20)
```

Step 4A: classification based on one orthophotomocaic

Step 4B: classification based on the combination of two orthophotomosaics

```
# 4B) classification scenario based on two-dates acquisitions
# generate all 2 dates combinations
pairs <- combn(ortho.names, 2,simplify=T)
# create a regular expression which is used for selecting appropriate
    metrics for each classification scenario
pairs <- paste(pairs[1,],pairs[2,], sep="|")
#creation of a matrix for storing result of the different classification
    scenarios
classif_2dates <- matrix(0,ncol=6, nrow=length(pairs), dimnames=list(pairs,
    c("overall out of bag error", "birches","common ash","English oak","
    poplars","sycamore maple")))

# run one random forest classification per pair
for (pair.num in c(1:length(pairs))){
  # select the metrics related to the combination of 2 orthophotomosaics by
      means of regular expression
  regex <- pairs[pair.num]
  selected.metrics <- crowns.metrics[, grep(regex, names(crowns.metrics))]
```

	birches	common ash	English oak	poplars	sycamore maple	class.error
birches	72	0	0	0	0	0
common ash	1	81	4	0	10	0.16
English oak	2	1	176	4	3	0.05
poplars	1	0	2	78	0	0.04
sycamore maple	3	9	22	0	108	0.24

Table 2: Result of line 27, step 4B. The confusion matrix of the optimal two-dates classification scenario (orthophotomosaics 3 and 4)

```
    # train the random forest
15  rf <- randomForest(crowns.metrics$SPECIES~ ., data=na.roughfix(selected.
        metrics), ntrees.shp=500,replace=T,na.action=na.omit)
    # save the random forest accuracy result in the result table
17  classif_2dates[pair.num, 2:6] <- round(100*rf$confusion[1:5,6], 0)
    classif_2dates[pair.num,1] <- round(100*mean(rf$confusion[,6]), 1)
19  cat(paste("End of training Random Forest classifier for multitemporal
        scenario ", regex, " ------------------------------ \n"))
  }
21
  # determine which pair is the optimal one and print a confusion matrix for
      this scenario
23 best_pair <- names(sort(classif_2dates[,1])[1])
   selected.metrics <- crowns.metrics[, grep(best_pair, names(crowns.metrics))]
25 rf <- randomForest(crowns.metrics$SPECIES~ ., data=na.roughfix(selected.
       metrics), ntrees.shp=500,replace=T,na.action=na.omit)
   cat(paste("The optimal two-dates combination for species classification is "
       , best_pair,sep=""))
27 print(rf)
```

Step 4B: classification based on the combination of two orthophotomosaics
